# Phosphate-independent utilization of phosphonoacetic acid as sole phosphorus source by a psychrophilic strain of *Geomyces pannorum* P15

**DOI:** 10.1007/s12223-014-0309-3

**Published:** 2014-02-26

**Authors:** Magdalena Klimek-Ochab

**Affiliations:** Department of Bioorganic Chemistry, Faculty of Chemistry, Wroclaw University of Technology, Wybrzeże Wyspiańskiego 27, Wrocław, 50-370 Poland

## Abstract

A psychrophilic fungal strain of *Geomyces pannorum* P15 was screened for its ability to utilize a range of synthetic and natural organophosphonate compounds as the sole source of phosphorus, nitrogen, or carbon. Only phosphonoacetic acid served as a phosphorus source for microbial growth in phosphate-independent manner. Substrate metabolism did not lead to extracellular release of inorganic phosphate. No phosphonate metabolizing enzyme activity was detectable in cell-free extracts prepared from *Geomyces* biomass pregrown on 2 mmol/L phosphonoacetic acid.

## Introduction

In the last few years, increased attention has been focused on a class of organisms called psychrophiles. Numerous organisms, prokaryotic but also eukaryotic, were found widely in natural and artificial cold environments (Georlette et al. 2004). They produce a variety of cold-adapted enzymes to carry out metabolism efficiently under cold conditions. A characteristic feature of many enzymes from psychrophiles is the correlation of high catalytic activity and low thermal stability at moderate temperatures, what allows to copy with the reduction of chemical reaction rates induced by low temperatures (Feller 2003). Naturally evolved psychrophilic enzymes require an improved flexibility of the structural components involved in the catalytic cycle, whereas other protein regions, if not implicated in catalysis, may or may not keep a high rigidity (Russell et al. 1998; Zhao et al. 2012). Various enzymes from psychrophilies and psychrotolerants have strong potential for various biotechnological applications, such as biotransformation, bioremediation, and so forth.

Organophosphonates are substances characterized by the presence of a carbon atom covalently bound to a phosphorus atom. This very stable bond occurs in a number of industrial, agricultural, medical, and household cleaning products (Nowack 2003), a fact that is raising increasing concerns due to their possible negative environment impact (Fenner et al. 2013). Several microorganisms of different origin have evolved the ability to utilize organophosphonates as a nutritional source of phosphorus (Kononowa and Nesmeyanowa 2002; McGrath et al. 2013; Sviridov et al. 2012; Forlani et al. 2011; Luo et al. 2011; Fan et al. 2012). The catabolism of organophosphonic acids by the carbon–phosphorus (CP) lyase pathway of *Escherichia coli* and numerous other bacteria requires the gene product of the 14-cistron *phn* operon, and both mechanism of CP bond cleavage and genetics of this process is still extensively studied (Kamat et al. 2011; Kamat et al. 2013; Hove-Jansen et al. 2011; He et al. 2011; Jochimsen et al. 2011).

To date, only a few enzymes catalyzing phosphonate hydrolysis have been purified and characterized (McGrath et al. 2013; Klimek-Ochab et al. 2006). In most cases, phosphonate utilization by bacteria was found to depend upon the phosphate status of the cell with several species where the enzymes expression seem independent of the availability of other P source (Ford et al. 2010; Kulakova et al. 2003; McMullan and Quinn 1994).

Phosphonate utilization by microbes may be an important and potentially underestimated component of P biogeochemical cycling (Falkowski et al. 2008; Martinez et al. 2009; Van Mooy et al. 2009).

Phosphonoacetic acid (PA) can serve as a model substrate for studying the microbial cleavage of P–C bond. The hydrolysis of PA to acetate and inorganic phosphate is catalyzed by PA hydrolase. This enzyme, isolated and characterized from some bacterial strains, is highly specific for its substrate and is neither repressed nor inhibited by the presence of high inorganic phosphate (Pi) level (McMullan and Quinn 1994). The protein from *Pseudomonas fluorescens* 23F is a Zn^2+^-dependent enzyme consisting of two identical subunits (McGrath et al. 1999). PA-hydrolase activity was also detected in cell-free extracts of two environmental isolates of *Curtobacterium* sp. and *Pseudomonas* spp.

Although bacterial metabolism of PA has been extensively studied (Fox and Mendz 2006), much less is known about the ability of fungi to utilize this substrate (Klimek-Ochab 2008). We previously reported that several *Penicillium* species are able to utilize PA as the only source of phosphorus. The enzyme providing fungi with such a capability was also PA hydrolase. However, it appeared to be different from the bacterial one (Klimek-Ochab et al. 2003; Forlani et al. 2006). The fungal PA hydrolase is a 43-kDa monomeric protein showing low affinity toward its substrate and high sensitivity to even mildly acidic pH values. Enzyme activity neither was required nor was stimulated by the presence of divalent cations (Klimek-Ochab et al. 2006).

The degradation process of phosphonic compounds was studied mainly in mesophilic microorganisms whereas little is known of the ability of extremophilic microbes to utilize these compounds. Indeed, the only documented reports describe thermophilic microbes (Obojska et al. 2002; Adams et al. 2008; Gomez-Garcia et al. 2011).

Degradation pathways have not been investigated in cold-adapted organisms. In this paper, we report for the first time that a psychrophilic fungal strain of *Geomyces pannorum* P15 was able to utilize PA as a source of phosphorus in the phosphate-independent manner.

## Materials and methods

### Microorganism

The psychrophilic fungal strain named P15 was a generous gift from Prof. Marianna Turkiewicz (Institute of Technical Biochemistry, Technical University of Lodz). This microorganism was identified in the DSMZ laboratory (Germany). Sequencing of the rDNA ITS fragment revealed a similarity of 99 % to *G. pannorum* NRBC 31776 and 98 % to *G. pannorum* ATCC 11501. The strain of P15 was therefore identified as *G. pannorum* (Link) Sigler & J.W. Carmich (MB 314399).

The filamentous fungus strain P15 used in this study is deposited in the Institute of Technical Biochemistry (Technical University of Lodz, Poland) collection of Antarctic microorganisms (http://snack.p.lodz.pl/ibtnew/en/research.htm). It was routinely maintained on Czapek–Dox agar, which provided profuse sporulation suitable for inoculum collection.

### Growth conditions

Degradation experiments were carried out with a modified Czapek liquid medium which consisted of: 30 g/L sucrose, 0.5 g/L MgSO_4_·7H_2_O, 0.5 g/L KCl, 2.64 g/L (NH_4_)_2_SO_4_, 0.01 g/L FeSO_4_·7H_2_O, and 0.5 g/L KH_2_PO_4_·7H_2_O, pH 7.2. When appropriate, the carbon or phosphate source was omitted and replaced by filter-sterilized PA; in the latter case, the medium was buffered with HEPES-KOH (5 mmol/L, pH 7.2). Cultures were grown either in 15-cm Petri dishes containing 50 mL medium or at 135 rpm in 250-mL Erlenmeyer flasks containing 100 mL medium, which were inoculated with a spore suspension in 0.05 % Triton X-100 to a density of 10^4^ spores per milliliter and incubated at 10 °C. Mycelium was harvested by vacuum filtration onto filter paper. To evaluate fungal growth, filters were dried for 48 h in an oven at 70 °C for dry-mass determination.

### Analytical methods

Phosphonoacetate residual concentration either in medium supernatants or in assay mixtures was determined by anion-exchange high-performance liquid chromatography (HPLC) as described previously (Klimek-Ochab et al. 2003). Inorganic phosphate was quantified colorimetrically by means of the green malachite acid dye assay (Forlani 1997). Protein concentration was measured by the method of Bradford (1976), using bovine serum albumin as the standard. All determinations and treatments were carried out at least in triplicate; reported values are means ± SD over replicates.

### Preparation of cell-free extract and hydrolase activity assay

Cultures grown to mid-exponential phase were harvested by centrifugation, and the mycelium was washed twice with ice-cold Tris–HCl buffer (50 mmol/L, pH 7.2), ground in a mortar with quartz sand, and further disrupted in a Cole Parmer Torbeo 36800 600-W sonicator with 4× cycles of 30 s sonication and 2 min cooling. Cell debris was removed by centrifugation at 20,000*g* for 15 min at 4 °C. The supernatant was dialyzed against Tris–HCl buffer (50 mmol/L, pH 7.2) overnight and loaded onto a DEAE–Sephacel column equilibrated with the same buffer. Retained proteins were eluted with a minimal amount of buffer containing 250 mmol/L NaCl, and the resulting extract was assayed toward PA hydrolase activity.

To measure phosphonoacetate hydrolysis, the reaction mixture contained 50 mmol/L Tris–HCl buffer, pH 7.2, 5 mmol/L PA, 1 mmol/L ZnSO_4_, and a limiting amount of enzyme in a final volume of 100 μL. After an appropriate incubation period (up to 60 min) at 10 °C, the inorganic phosphate released was quantified colorimetrically. Routinely, the hydrolase activity was expressed as the amount of phosphate released per second per milligram of protein.

## Results

### Phosphonoacetate utilization by *Geomyces pannorum* P15

**Fig. 1:**
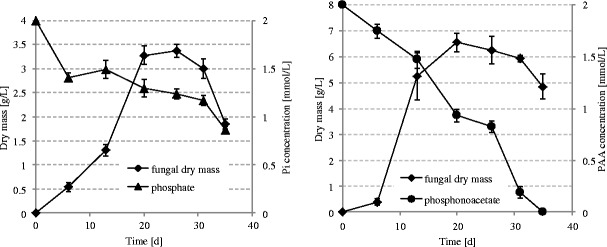
Growth of *G. pannorum* P15 in media containing inorganic phosphate or phosphonoacetic acid as P sources and fungal utilization of both substrates. Data are means ± SD of three replication

Several psychrophilic fungal isolates were examined for their ability to utilize PA for cell purposes. Among them, a strain of *G. pannorum* P15 was found to grow in modified Czapek liquid medium containing the PA at 2-mmol/L concentration as the only source of phosphorus. Uninoculated controls showed no significant decomposition of the substrate. No phosphate release to the culture medium occurred during growth of *G. pannorum* on modified medium. Quite interestingly, biomass yields were much higher than those in complete medium (Fig. 1). In contrast no fungal growth was evident if the substrate was supplied as a carbon source.

Surprisingly, only the partial depletion of inorganic phosphate from the culture medium was observed in the case of non-modified medium, whereas complete utilization of PA occurred when phosphonate served as a P source. ^31^P NMR analysis of fungal cell-free extracts showed the presence of PA (*δ* = 16.52 ppm), proving the uptake and accumulation of the phosphonic substrate in the fungal cell.

Our study also found that PA utilization by psychrophilic isolate strongly depends on the temperature of fungal cultivation. HPLC analysis of culture supernatants clearly demonstrated that PA was removed most effectively at 10 °C (Table 1).

**Table 1:** Utilization of PA by *Geomyces pannorum* P15 at different temperatures of cultivation

Temperature of growth [°C]	Percent of substrate utilization
4	35
10	52
15	12
20	2

2 mmol/L of PAA as the sole source of P. Final concentration of PAA was evaluated 20 days after inoculation. Data are means ± SD over three replications

The effect of rising concentration of PA in culture medium on *G. pannorum* P15 growth was also studied. The concentration of PA up to 15 mmol/L stimulated the biomass production; however, no influence was observed when the substrate concentration exceeded this value (Table 2).

**Table 2:** Utilization of PA by *G.pannorum* P15 cultivated on Czapek–Dox medium containing various concentrations of organophosphonate as the only source of phosphorus

PA concentration in the cultivation medium [mmol/L]	mmol of PA/g of biomass	Biomass yield [g/L]
2	0.27 ± 0.08	7.72 ± 0.25
5	0.55 ± 0.02	9.26 ± 0.12
10	0.62 ± 0.11	13.01 ± 0.26
15	0.93 ± 0.09	14.26 ± 0.18
20	0.94 ± 0.02	12.86 ± 0.31

Final biomass and concentration of substrate were evaluated 35 days after inoculation. Data are means ± SD over three replication

**Fig. 2:**
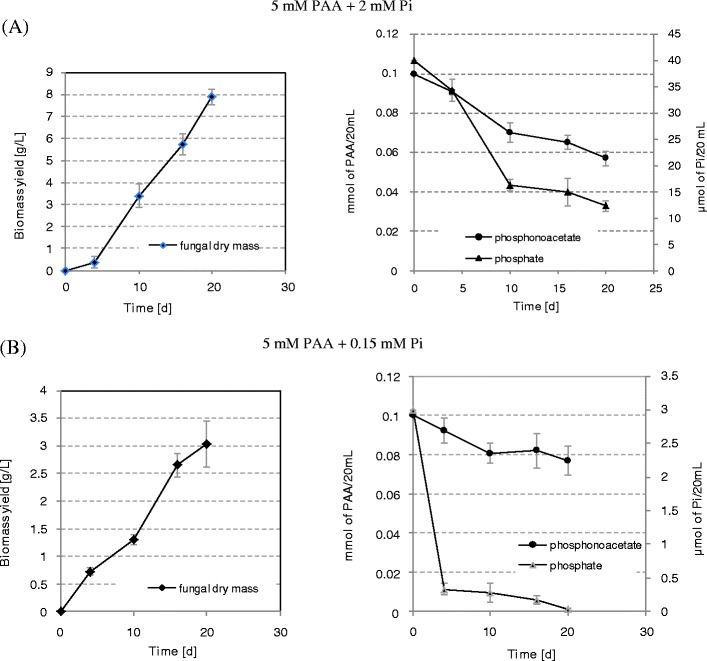
Growth of *Geomyces pannorum* P15 in media containing inorganic phosphate and phosphonoacetic acid as P source. The residual concentration of both substrates in the broth supernatants was also evaluated. Data are means ± SD of three replication. **a** 5 mM PAA + 2 mM Pi. **b** 5 mM PAA + 0.15 mM Pi

In the next step, the interference of Pi with PA utilization was extensively studied. Biomass yield and substrate utilization by fungal isolate were determined in media containing different combination of PA and Pi. Results are showed in Fig. 2.

Surprisingly, when the concentration value of inorganic phosphate was 2 mmol/L and the PA 5 mmol/L (in the cultivation medium, Fig. 2a), the concentration of both substrates decreased significantly with time of cultivation and biomass yield as well as growth kinetics were comparable with those obtained when psychrophilic strain was cultivated in medium containing only phosphonate substrate (Fig. 1). If inorganic P was available, but at very low concentrations (0.15 mmol/L), it was removed completely, with a very high rate during the first 5 days of cultivation (Fig. 2b), whereas PA utilization in such conditions do not exceed 25 % of started value (5 mmol/L, Fig. 2b). It is worth to stress that biomass yield obtained in these conditions was also much lower than in the case discussed above, see Fig. 2a.

In order to better characterize the ability to degrade carbon-to-phosphorus bond by the fungal isolate, a series of structurally diverse phosphonates were tested. *N*,*N*-bis(phosphonomethyl)glycine, 2-aminoethylphosphonic acid, *N*-phosphonomethylglycine, phenylphosphonic acid, and aminobenzylphosphonic acid failed to serve as a P source. Surprisingly, examined *Geomyces* strain was able to utilize methylphosphonic acid, 1-amino-3-methylbutylphosphonic acid, and *N*-(phosphonomethyl)iminodiacetic acid supplied as a sole source of phosphorus with biomass yields exceeding 5 g/L after 20 days of cultivation. Degradation of phosphonic substrates utilized by fungal strains was confirmed by ^31^P NMR analysis and no phosphonic products of degradation by *Geomyces* were detected during the time of experiment.

#### Enzymatic activity of cell-free extract toward PA

Since PA contains carboxyl group adjacent to its CP bond, it would be of particular interest to determine if the enzymatic mechanism of metabolism proceeds via hydrolase or CP lyase-like reaction. Many attempts have been undertaken to obtain enzymatically active crude extract of psychrophilic *Geomyces* strain. To establish the most efficient method of fungal cell lysis, fresh or frozen cells of psychrophiles were subjected to various disruption protocols (grounding in a mortar with liquid nitrogen, sonications, chemical treatment, and osmotic shock, data not shown). The mechanical method sonication appeared to be the only one effective for disintegration of fungal cell-wall, and the level of released proteins was about 0.6 mg/mL of crude extract. Unfortunately, despite a number of attempts, no cell-free activity of enzyme(s) toward PA could be obtained.

## Discussion

The present study proves for the first time that psychrophilic fungal strain *G. pannorum* P15 can cleave CP bonds and utilize phosphonacetic acid for growth. The degradation of substrate in fungal culture was complete only when PA served as a sole phosphorus source and appeared to be a temperature-dependent process. Raising the substrate concentration in the cultivation medium up to 15 mmol/L stimulated the fungal biomass production.

The acquisition of adequate supplies of P is a priority for all living cells. Pi is the preferred P source for cellular growth, and under conditions of Pi starvation, a number of gene systems whose products are involved in its acquisition and assimilation are induced (Quinn et al. 2007). It is worth to note that studied microorganism isolated from the synthetic phosphonate-intact environments possess some enzymatic mechanisms involved in the utilization of P–C-containing compounds. In the cold ecosystems, Pi concentration is sometimes in the subnanomolar range (Dyhrman and Haley 2006) and it is often, and increasingly (Karl 2000), the limiting nutrient. In such circumstances, the ability to access less readily available forms of organically complexed P would provide a competitive advantage for survival. The facility for PA utilization by fungal cells seemed to be a characteristic feature of adaptation of particular strain of *Geomyces* because among three tested strains of *G. pannorum*, only the isolate P15 was able to grow on this substrate used as phosphorus source in the cultivation medium. However, research has shown that laboratory cultures of *G. pannorum* isolated from various environments may exhibit extreme differences in morphology and physiology. In fact, the limits of cold adaptation of a particular isolate can vary depending on the source of isolation, even though the isolates are genetically identical (Kochkina et al. 2007).

The degradation of PA by psychrophilic strain has been found to be independent on the phosphate status of the cell. In the case of P15 strain, the utilization of P–C substrate in the presence of inorganic phosphorus in the cultivation medium exhibited quite unexpected course. If both phosphorus sources, namely, the inorganic phosphate and PA, are present in the medium at the sufficient levels, the utilization of phosphate occurs concomitantly with PA degradation. But, if inorganic phosphate was available at a very low level, the utilization of PA by *Geomyces* stopped after phosphate had been completely taken up by the cells. These results are distinctly different from those obtained previously in our laboratory with *Penicillium* strains, which were able to mineralize PA only in a phosphate-sensitive manner (Forlani et al. 2006).

Possibly, in the *Geomyces* cells, two independent transport systems for PA uptake exist. In the presence of inorganic phosphorus, phosphate transport into the cell is concomitant with PA transport across the cell envelopes. In the absence of Pi, a different type of PA transport occurred. Both transport mechanisms cannot be active at the same time. Transport processes in fungi have been studied in only in the case of few different organisms with *Saccharomyces cerevisiae* and *Neurospora crassa* being the most frequently chosen experimental subjects and less often with *Aspergillus nidulans*. However, the significant differences in transport properties determined among these and other fungi indicate the great variety of physiological adaptations that have evolved among them. Considering the intimate contact that hyphae make with the environment and many different conditions to which fungi are adopted, it is not surprising that variation in transport system was found.

Mesophilic microorganisms utilizing the PA for cell purposes were found to possess active phosphonoacetate hydrolase in the cells. Different forms of this enzyme have been identified within the environmental microflora, as the enzyme occurs in both Gram positive and Gram negative bacteria as well as in filamentous fungi (Kononowa and Nesmeyanowa 2002; Forlani et al. 2006). Unfortunately, despite many attempts, no cell-free extract activity toward PA has been demonstrated in the case of psychrophilic *Geomyces* strain. The lack of detectable hydrolase activity can suggest presence of either cold-tolerant CP lyse-like enzymes or functioning of completely new enzyme(s) involved in PA mineralization by psychrophilic cells. It was generally believed that only one type of CP lyase exists, but the suggestion of Kertesz et al. (1991) that variant forms of the enzyme may occur has been confirmed (White and Metcalf 2004; McGrath et al. 2013). Further indication of the diversity of the enzyme system has come from in silico analysis which has identified homologous (occasionally multiple) CP lyase gene clusters across a wide range of prokaryotic groups; these show considerable structural and compositional variation (Huang et al. 2005).
